# Serum levels of Interleukin-10, Interleukin-6, and leptin in patients with *Mycobacterium leprae*–Helminth Co-Infections

**DOI:** 10.1186/s12879-025-11752-2

**Published:** 2025-11-14

**Authors:** Hendra Gunawan, Risa Miliawati Nuruh Hidayah, Nurul Hidayah, Reti Hindritiani, Kartika Ruchiatan, Pati Aji Achdiat, Chaerani Pratiwi Firdaus

**Affiliations:** Department of Dermatology and Venereology, Faculty of Medicine, Universitas Padjadjaran - Dr. Hasan Sadikin General Hospital, Jl. Pasteur No. 38, Bandung, 40161 West Java Indonesia

**Keywords:** Helminth infection, IL-6, IL-10, Leprosy, Leptin

## Abstract

**Introduction:**

Leprosy remains a public health problem in several countries, including Indonesia, due to its surrounding disability, stigma, and discrimination. In leprosy, compromised immune responses frequently affect the occurrence of helminth infections, which are known to contribute to a number of diseases by upregulating T-helper 2. Elevated levels of interleukin (IL)-10, IL-6, and leptin have been associated with helminth infection and leprosy, respectively.

**Purposes:**

This study aimed to measure the serum levels of IL-10, IL-6, and leptin in leprosy patients with and without helminth co-infections.

**Patients and methods:**

A cross-sectional design was used for this observational clinical study. A total of 32 leprosy patients who fulfilled our inclusion and exclusion criteria were recruited. The quantification of IL-10 and IL-6 levels was performed using serum samples with a quantitative sandwich enzyme-linked immunosorbent assay method. Additionally, the quantification of leptin serum was also carried out. The helminth’s eggs were examined using the Kato-Katz technique.

**Results:**

This study revealed that helminth co-infection was present in five out of 32 (15.6%) leprosy patients. The types of helminth found in leprosy patients with helminth infections were *Ascaris lumbricoides* and *Trichuris trichiura*, with a mild intensity of infection. For leprosy with helminth infection, the mean IL-10, IL-6, and leptin serum levels were 86.43 pg/ml ± 4.34 pg/ml, 447.42 pg/ml ± 122.15 pg/ml, and 19,272.85 pg/ml ± 4,065.23 pg/ml, respectively. Meanwhile, the mean IL-10, IL-6, and leptin serum levels for leprosy without helminth infection were 72.97 pg/ml ± 14.69 pg/ml, 161.77 pg/ml ± 63.98 pg/ml, and 14,649.07 pg/ml ± 2,002.66 pg/ml, respectively. Based on an independent t-test, the difference in serum levels of IL-10 and IL-6 in leprosy patients with and without helminth infections was *p* = 0.001 and *p* = 0.0001, respectively. However, based on the Mann-Whitney test, the serum level of leptin between leprosy patients with and without helminth infections was *p* = 0.062.

**Conclusion:**

The proportion of helminth infection in leprosy patients is 15.6%. Leprosy patients with helminth infections have considerably greater serum levels of IL-10 and IL-6, suggesting that helminth infection influences leprosy patients’ immunological responses.

## Introduction

Leprosy is a chronic granulomatous infectious disease caused by *Mycobacterium leprae* (*M. leprae*), a Gram-positive, obligate intracellular, and acid-resistant bacterium that infects macrophages and Schwann cells [1]. The transmission of *M. leprae *is suspected to occur through the upper respiratory tract and skin [1, 2], where they will activate the innate and acquired immune systems in response to their invasion of the host [3]. Based on the data from the World Health Organization (WHO), there were 174,087 new cases of leprosy from 182 countries in six regions in 2022. Globally, India is at the top of the list with 103,819 new leprosy cases, and Indonesia is number three with 12,441 cases [4]. Indonesia reported an average of 0.45 new leprosy cases per 100,000 people in 2021 [5]. In 2022, there were 1,721 new leprosy cases in West Java, Indonesia [6]. 

The occurrence of bacterial, parasitic, and helminth infections in leprosy patients is influenced by the immune system of the patients [7]. The immune response in leprosy patients, particularly the T-helper (Th)1/Th2 imbalance, influences susceptibility to other infectious diseases. Tuberculoid leprosy’s strong Th1 response protects against intracellular pathogens (e.g., viruses, some bacteria) but weakens defense against extracellular pathogens like helminths. Otherwise, in lepromatous leprosy, the dominant Th2 responses increase susceptibility to intracellular pathogens but may exacerbate helminth infections, as helminths thrive in a Th2-rich environment [8]. Reports indicate that nearly 25% of the world’s human population suffers from helminth infections [9]. Nematodes and platyhelminthes are the two major phyla of helminths. Soil-transmitted helminths (STH) belong to the group of nematodes [10]. In some developing countries, helminth infections are still a major issue with a relatively high mortality and morbidity rate [9]. In Indonesia, helminth infection remains a public health problem due to its relatively high prevalence of 45–65% and up to 80% in some regions with poor sanitation [11]. 

T-helper 2 cells are a subset of T-cells involved in helminth infections and leprosy that produce inflammatory mediators [1, 12]. Patients with lepromatous leprosy exhibit a Th2 response, producing significant amounts of antibodies and interleukin (IL)-10 [1]. Invasion of peripheral nerves by *M. leprae *also causes an immune response in the host, as shown by an increase in the production of IL-6 [13]. Moreover, studies have shown that *M. leprae* induces inflammation by stimulating leptin release, for example, through bacterial lipopolysaccharide (LPS) stimuli [14]. 

In helminth infections, the activation of alternative pathway macrophages will increase regulatory T cell (Treg) cell secretion, characterized by an increased IL-10 level [12]. Therefore, IL-10 plays a role in increasing the level of cytokines and proteins produced by adipose tissue, such as IL-6 and leptin [15]. Serum levels of IL-10, IL-6, and leptin are known to be raised in leprosy patients [1, 13, 14] and in helminth infection patients, respectively [12, 15]. 

Helminth infections may affect the host immune response and may consequently influence the progression of leprosy [7]. The aim of this study was to investigate the impact of helminth infection on the immune responses of leprosy patients. This study was the first in Indonesia to measure inflammatory cytokines, specifically serum levels of IL-10, IL-6, and leptin, with a focus on two major neglected tropical diseases.

## Materials and methods

### Study sites and participants

Our study participants were leprosy patients who attended Dr. Hasan Sadikin General Hospital Bandung from December 2021 to March 2022. The Medical Ethics Committee of the Faculty of Medicine, Universitas Padjadjaran has approved this study (project number LB.02.01/X.6.5/298/2021). We recruited our participants by taking their medical histories according to our selection criteria.

The inclusion criteria for this study were patients who have been diagnosed with paucibacillary (PB) or multibacillary (MB)-type leprosy based on cardinal signs, including new patients, those who were currently on medications or released from treatment (RFT), leprosy patients who have or have not experienced leprosy reactions, and those who were ≥ 18 years old and have a normal body mass index (BMI).

The exclusion criteria were those who had taken anthelmintic drugs three months prior to the stool and blood examinations; patients who had taken laxatives and hypertonic fluids for the past 14 days before the stool and blood examinations; those who were pregnant, breastfeeding, or having periods; and patients who had chronic systemic diseases, such as diabetes mellitus, tuberculosis, and human immunodeficiency virus (HIV)/acquired immunodeficiency syndrome (AIDS).

## Field and laboratory procedures

After the diagnosis of leprosy was established, the researcher filled out medical records that included the patient’s identity, history, physical examination, working diagnosis, and therapy. Subsequently, their serum levels of IL-10, IL-6, and leptin were then measured, and samples of their stool were taken to check for the presence of helminth eggs.

IL-10, IL-6, and leptin serum levels were measured using a human IL-10 enzyme-linked immunosorbent assay (ELISA) plate (Elabscience, Cat: E-EL-H6154), a human IL-6 ELISA plate (Elabscience, Cat: E-EL 128 H6154), and a human LEP ELISA plate (Elabscience, Cat: E-EL-H6017), respectively, according to the manufacturer’s instructions (USA). All participants self-collected their stool samples for the helminth egg examination. The Kato-Katz smear technique was performed one time in every sample. The sieved feces sample (approximately 41.7 mg) was placed on a glass slide, then was covered with a piece of cellophane soaked in glycerol to ‘clear’ the fecal material from around the eggs. The eggs were then counted under the microscope, and the count was expressed in per gram of feces [16]. A parasitologist carried out this procedure. The intensity of helminth infections was classified as mild, moderate, and heavy based on egg per gram (epg). The participants were then divided into two groups: those with helminth-positive and helminth-negative. The helminth-positive participants were subsequently treated with antihelminthic therapy.

### Statistical analysis

All of our data were analyzed using the Statistical Package for the Social Sciences version 17. All of the quantitative variables were estimated using measures of central location (mean, median, and interval). An independent t-test was utilized to determine the comparison of serum IL-10 and IL-6 levels, while a Mann-Whitney test was used to establish the comparison of serum leptin levels in leprosy patients with and without helminth co-infection.

## Results

The study was conducted on 32 leprosy patients; five of them (15.6%) had helminth infections, and 27 of them (84.4%) were without helminth infections. Leprosy patients were most commonly found in the age group of 18–45 years (14 patients), followed by 13 patients in the age group of 46–65 years. In leprosy cases without helminth infection, the patients’ age ranged from 18 to 72 years with an average of 47 years, whereas in leprosy cases with helminth infection, the patients’ age ranged from 30 to 64 years with an average of 45 years. Most of the leprosy patients in our study were male (78.1%) who had finished their primary school education (46.9%). In this study, 31.3% of leprosy patients worked as farmers (Table 1).

**Table 1:** General characteristics of study participants

Variables	Helminth Infection				Total	
	Negative		Positive			
	*n* = 27	%	*n* = 5	%	*n* = 32	%
**Age**						
18–45 years old	11	40.7	3	60.0	14	43.8
46–65 years old	11	40.7	2	40.0	13	40.6
> 65 years old	5	18.6	0	0.0	5	15.6
**Sex**						
Male	21	77.8	4	80.0	25	78.1
Female	6	22.2	1	20.0	7	21.9
**Education**						
Primary School	13	48.2	2	40.0	15	46.9
Junior High School	2	7.4	1	20.0	3	9.4
Senior High School	9	33.3	2	40.0	11	34.3
Bachelor’s Degree	3	111	0	0.0	3	9.4
**Occupation**						
Farmer	8	29.7	2	40.0	10	31.3
Enterpreneur	7	25.9	0	0,0	7	21.9
Housewife	3	11.1	1	20.0	4	12.5
Student	3	11.1	0	0,0	3	9.4
Unemployed	2	7.4	0	0,0	2	6.3
Government employees	1	3.7	0	0,0	1	3.1
Laborer	1	3.7	0	0,0	1	3.1
Fisherman	0	0.0	1	20.0	1	3.1
Workshop worker	1	3.7	0	0,0	1	3.1
Driver	1	3.7	0	0,0	1	3.1
Garden	0	0.0	1	20.0	1	3.1
**BMI**						
Underweight	0	0.0	0	0.0	0	0.0
Normoweight	27	100	5	100	32	100
Overweight	0	0.0	0	0.0	0	0.0
Obese	0	0.0	0	0.0	0	0.0

Table 2 provides the clinical summary of all of the study participants. The study found the most common types of leprosy to be MB-type leprosy (WHO classification) and borderline leprosy (BL) type leprosy (Ridley-Jopling classification), with a prevalence of 90.6% and 40.6%, respectively. The duration of prednisone therapy in patients who experienced leprosy reactions was mostly more than 12 weeks (37.5%), with an average duration of administration of 15.7 weeks and the longest duration of 24 weeks.

**Table 2:** Clinical overview of study participants

Variable	Helminth Infection				Total	
	Negative		Positive			
	*n* = 27	%	*n* = 5	%	*n* = 32	%
**Leprosy Classification** **WHO**						
Paucibacillary	2	7.4	1	20.0	3	9.4
Multibacillary	25	92.6	4	80.0	29	90.6
**Ridley Jopling**						
TT	0	0.0	0	0.0	0	0.0
BT	2	7.4	1	20.0	3	9.4
BB	6	22.2	2	40.0	8	25.0
BL	13	48.1	0	0.0	13	40.6
LL	6	22.2	2	40.0	8	25.0
**Medication Status**						
On MDT therapy	19	70.4	5	100.0	24	75.0
Release from treatment	8	29.6	0	0	8	25.0
**Reaction Status**						
With Reaction	19	70.4	3	60.0	22	68.7
Without Reaction	8	29.6	2	40.0	10	31.3
**Prednisone Therapy Duration**						
** 12 weeks**						
RR	6	22.3	1	20.0	7	21.9
ENL	2	7.4	0	0.0	2	6.3
** > 12 weeks**						
RR	2	7.4	0	0.0	2	6.3
ENL	8	29.6	2	40.0	10	31.2
**Without therapy**						
Mild RR	1	3.7	0	0.0	1	3.1
Without Reaction	8	29.6	2	40.0	10	31.2

Most leprosy patients with helminth infections were MB-type (80%). The types of helminth found in leprosy patients with helminth infections were *Ascaris lumbricoides* and *Trichuris trichiura*, with a mild intensity of infection. The average number of eggs of the *Ascaris lumbricoides* helminth was 96/epg, while the average number of eggs of the *Trichuris trichiura* helminth was 120/epg (Table 3).

**Table 3:** Clinical characteristics of leprosy patients with helminth infection

No	Leprosy Classification (WHO/Ridley-Jopling)	Leprosy Reaction Classification	Helminth Species	Number of Helminth’s eggs (epg)	Intensity of Helminth Infection
1.	PB/BT	RR	*Ascaris lumbricoides*	120	Mild
2.	MB/LL	ENL	*Trichuris trichiura*	144	Mild
3.	MB/LL	Without reaction	*Ascaris lumbricoides*	72	Mild
4.	MB/BL	Without reaction	*Trichuris trichiura*	96	Mild
5.	MB/BL	ENL	*Trichuris trichiura*	120	Mild

Based on the results of statistical analysis using independent t-tests between leprosy patients with and without helminth infection, a p-value of 0.001 (*p* < 0.05) was obtained for serum IL-10 levels and 0.0001 (*p* < 0.05) for serum IL-6 levels. These results showed a statistically significant difference in serum IL-6 and IL-10 levels between leprosy patients with and without helminth infections. However, the Mann-Whitney test revealed a p-value of 0.062 (*p* > 0.05) for serum leptin levels between leprosy patients with and without helminth infections. These results revealed statistically insignificant differences in serum leptin levels between leprosy patients with and without helminth infections (Table 4).

**Table 4:** Comparison of IL-10, IL-6, and leptin serum levels between leprosy patients with and without helminth infection

Variables	Helminth Infection		*p* value
	Negative	Positive	
	*n* = 27	*n* = 5	
**IL-10 level (pg/ml)**			0.001
Mean ± Std	72.97 ± 14.69	86.43 ± 4.34	
Median	82.06	83.93	
Interval	36.43–92.66	83.16–93.08	
**IL-6 level (pg/ml)**			0.0001
Mean ± Std	161.77 ± 63.98	447.42 ± 122.15	
Median	140.80	391.11	
Interval	46.74–294.05	308.45–583.55	
**Leptin level (pg/ml)**			0.062
Mean ± Std	14649.07 ± 2002.66	19272.85 ± 4065.23	
Median	14366.23	18351.92	
Interval	10735.30–19043.47	13731.40–24626.28	

## Discussion

In this study, the average IL-10, IL-6, and leptin serum levels in leprosy patients with helminth infections were higher than those without helminth infection. IL-6 is a pro-inflammatory cytokine that has a role in inhibiting the secretion of IL-1 and tumor necrosis factor (TNF)-α secretion, as well as activating IL-10, which is an anti-inflammatory cytokine [17]. Toll-like receptors (TLR) 1, TLR2, TLR4, dendritic cell-specific intercellular adhesion molecule-3-grabbing non-integrin (DC-SIGN), and CD163 mediate the interaction between macrophages and *M. leprae *in leprosy, which increases IL-6 and IL-10 secretion. PB-type leprosy more commonly exhibits TLR1 and TLR2, while MB-type leprosy more commonly exhibits TLR4 [1]. DC-SIGN, Raf-1 activation, and acetylization of nuclear factor-kappa B (NF-kB) p65 after TLR activation mediate the increase in IL-10 level in dendritic cells [1]. TLR4 primarily mediates the increase in IL-6 production by *M. leprae*, followed by a rise in TNF-α and interferon-induced protein 10 (IP-10) [1, 18]. 

Based on a study conducted by Madan et al., [17] it was known that the average of IL-10 serum levels was significantly higher in leprosy patients compared to the non-leprosy group. Researchers found that MB-type leprosy had a more elevated level than PB-type [17]. These findings were aligned with the study conducted by Sutedja et al. [19] and Nada et al. [20] In a study conducted by Belgaumkar et al., [21] they revealed that the average IL-6 serum levels in leprosy-type mid-borderline (BB), BL, and lepromatous leprosy (LL) patients were significantly higher compared to the non-leprosy group.

In leprosy, liposaccharides contained in the outermost layer of the cell wall of *M. leprae* can stimulate the secretion of leptin [14]. An increased level of leptin can also affect the incidence of neuropathy. Leptin will activate signal transducer and activator of transcription (STAT) 3, which is a transcription factor in cells that helps the process of cell proliferation, migration, and apoptosis. Activation of STAT3 will stimulate macrophages and cause peripheral nerve damage [22]. Saraya et al. reported that the average leptin serum level in leprosy patients was significantly higher than in the non-leprosy group [14]. As far as the authors’ knowledge, there were only a few studies reporting serum leptin levels in leprosy patients.

In helminth infections, Treg cells drive an increase in IL-10 levels as a result of macrophage activation through alternative pathways [10]. Additionally, antigens in helminths can also stimulate Th2 cytokines, such as IL-4, IL-5, and IL-10 [23]. The latter plays a role in increasing levels of cytokines and proteins, such as IL-6, leptin, and TNF-α. Helminth infections in the intestines affect the concentration of leptin in the blood [15]. This is due to leptin’s role in preventing epithelial apoptosis and facilitating tissue repair, which is part of the intestinal mucosa’s defense mechanism against pathogens [24]. 

Nurhayati et al. [12] found that the STH-infected group had higher IL-10 serum levels than the non-STH-infected group, with a median of 278 pg/ml and 204 pg/ml, respectively [12]. Several studies also revealed similar results, such as the studies conducted by Goddey et al., [25] Olagbegi et al., [26] Goncales et al., [27] Yahya et al., [28] and Karul et al., [29] that reported a higher average of IL-10, IL-6, or leptin serum levels in the STH-infected group compared to the non-STH-infected group.

Based on the results of the statistical analysis in this study, there were significant differences in IL-10 (*p* = 0.001) and IL-6 (*p* = 0.0001) serum levels between leprosy patients with and without helminth infections. In addition, the 80% of leprosy patients who contracted helminth infections were of the MB-type. Helminth co-infection in leprosy is known to facilitate the growth and spread of *M. leprae* by upregulating Th2 cytokines or Tregs [7]. In leprosy, Th2 stimulates the secretion of IL-10 and is responsible for inhibition of macrophage function, suppression of cell-mediated immunity, and antibody production [21]. *M. leprae* will also invade peripheral nerves and multiply in Schwann cells, causing nerve cell damage. The body’s immune response to protect nerve damage is characterized by increased secretion of IL-6 levels [13]. In helminth infections, antigen proteins activate Th2 to stimulate the secretion of IL-4 and IL-13, which will stimulate the secretion of Tregs and IL-10 [12, 23]. IL-10 contributes to an increased IL-6 serum level [15]. Intestinal tissue damage due to helminth infections will also increase the secretion of IL-10 and IL-6 [15]. There is an association between helminth infection and leprosy through altering the host immune response against *M. leprae.* The Th2-dominated immune response in MB-type leprosy creates an environment that favors helminth survival and growth, potentially worsening helminth infections in these patients [7]. The increased Th2 regulation in leprosy patients with helminth infections is considered to be the cause of raised IL-10 and indirectly also increases IL-6 levels in leprosy with helminth infections compared to those without helminth infections.

Based on our data, there was no significant difference in leptin serum levels between leprosy patients with and without helminth infections (*p* = 0.062). An intestinal parasitic infection may influence the concentration of leptin in the blood. Patients who had multiple parasitic infections were more likely to had higher leptin concentrations than those who were parasite-free. Additionally, a higher concentration of leptin was associated with specific intestinal parasites. Each type of intestinal parasitic infection has been attributed to leptin concentration [15]. In this study, the helminths identified were *Ascaris lumbricoides* and *Trichuris trichiura* with mild intensity. There were no multiple infections in a single individual. We suspected this to be one of the reasons for the lack of significant difference in serum leptin levels between the helminth-infected group and the non-infected group.

## Conclusion

Interleukin-10 and IL-6 play a role in leprosy patients with helminth co-infections, which indicates that there is an influence of helminth infection on the immune responses of leprosy patients. Monitoring these cytokines could help identify patients at risk of immune dysregulation and guide targeted interventions. However, further studies are still required to investigate the inflammatory markers in leprosy patients with helminth infection.

### Limitations

1. We only conducted the stool examination once, rather than serially or multiple times. Serial and multiple stool sample collections are known to increase the likelihood of positive findings of helminth eggs in fecal examinations.
2. The relatively small number of patients in our study limited our findings.

## Data Availability

Data supporting this study’s findings are available from the corresponding author upon reasonable request.

## Abbreviations

AIDS: Acquired immunodeficiency syndrome
BB: Mid-borderline
BL: Borderline leprosy
BMI: Body mass index
DC-SIGN: Dendritic cell-specific intercellular adhesion molecule-3-grabbing non-integrin
ELISA: Enzyme-linked immunosorbent assay
HIV: Human immunodeficiency virus
IL: Interleukin
IP-10: Interferon γ-induced protein 10
LL: Lepromatous leprosy
LPS: Lipopolysaccharide
MB: Multibacillary
*M. leprae*: *Mycobacterium leprae*
NF-kB: Nuclear factor-kappa B
PB: Paucibacillary
RFT: Released from treatment
STAT: Signal transducer and activator of transcription
STH: Soil-transmitted helminths
Th: T-helper
TLR: Toll-like receptor
Treg: Regulatory T cell
TNF: Tumor necrosis factor
WHO: World Health Organization
